# Chronic P‐glycoprotein inhibition increases the brain concentration of escitalopram: potential implications for treating depression

**DOI:** 10.1002/prp2.190

**Published:** 2015-10-26

**Authors:** Fionn E. O'Brien, Gerard M. Moloney, Karen A. Scott, Richard M. O'Connor, Gerard Clarke, Timothy G. Dinan, Brendan T. Griffin, John F. Cryan

**Affiliations:** ^1^ APC Microbiome InstituteUniversity College CorkCorkIreland; ^2^ Pharmacodelivery GroupSchool of PharmacyUniversity College CorkCorkIreland; ^3^ Department of Anatomy & NeuroscienceUniversity College CorkCorkIreland; ^4^ Department of PsychiatryUniversity College CorkCorkIreland; ^5^ Present address: UCL School of PharmacyUniversity College LondonLondonUnited Kingdom; ^6^ Present address: Department of Pharmacology and Systems TherapeuticsIcahn School of MedicineMount Sinai HospitalNYUSA

**Keywords:** Antidepressant, antidepressant augmentation, blood–brain barrier, escitalopram, P‐glycoprotein, treatment‐resistant depression

## Abstract

Recent preclinical studies have revealed a functionally important role for the drug efflux pump P‐glycoprotein (P‐gp) at the blood–brain barrier in limiting brain levels and thus antidepressant‐like activity of certain antidepressant drugs. Specifically, acute administration of P‐gp inhibitors, such as verapamil and cyclosporin A (CsA), has been shown to augment brain concentrations and functional activity of the antidepressant escitalopram in rodents. However, depression is a chronic disorder and current treatments require prolonged administration to elicit their full therapeutic effect. Thus, it is important to investigate whether acute findings in relation to P‐gp inhibition translate to chronic paradigms. To this end, the present study investigates whether chronic treatment with the P‐gp inhibitor verapamil and the antidepressant escitalopram results in enhanced brain distribution and antidepressant‐like effects of escitalopram. Verapamil (10 mg·kg^−1^ i.p.) and escitalopram (0.1 mg·kg^−1^ i.p.) were administered once daily for 22 days. On the final day of treatment, brain regions and plasma were collected for analysis of cortical and plasma escitalopram concentrations, and to determine the hippocampal expression of genes previously reported to be altered by chronic antidepressant treatment. Verapamil treatment resulted in a greater than twofold increase in brain levels of escitalopram, without altering plasma levels. Neither gene expression analysis nor behavioral testing revealed an augmentation of responses to escitalopram treatment due to verapamil administration. Taken together, these data demonstrate for the first time that P‐gp inhibition can yield elevated brain concentrations of an antidepressant after chronic treatment. The functional relevance of these increased brain levels requires further elaboration.

**Abbreviations**
ANOVA: analysis of variance
BBB: blood–brain barrier
BDNF: brain‐derived neurotrophic factor
CREB: cAMP‐responsive element‐binding protein
EGR1: early growth response protein 1
NGFI‐B: nerve growth factor IB
P‐gp: P‐glycoprotein
SERT: serotonin transporter
SSRI: selective serotonin reuptake inhibitor
TST: tail suspension test

## Introduction

Increasing data have revealed that the multidrug efflux transporter P‐glycoprotein (P‐gp), expressed at the blood–brain barrier (BBB), restricts brain levels of several clinically important antidepressant drugs, thereby potentially contributing to the high prevalence of treatment failure (Uhr et al. 2008; O'Brien et al. 2012a,b, 2013a). Moreover, we have recently demonstrated that acute inhibition of P‐gp by verapamil enhances the behavioral effects of the antidepressant escitalopram in the tail suspension test (TST) (O'Brien et al. 2013b), one of the most widely used and well‐validated animal models to assess antidepressant‐like activity (Cryan et al. 2005). Taken together, these findings raise the possibility that adjunctive treatment with a P‐gp inhibitor may represent a potentially beneficial augmentation strategy in treatment‐resistant depression.

Most studies investigating the effect of P‐gp on antidepressant distribution into the brain have focused on acute drug administration (O'Brien et al. 2012b). While a limited number of research articles have reported that brain levels of certain antidepressants are elevated in P‐gp knockout mice relative to wild‐type controls after subchronic (10–11 days) treatment (Grauer and Uhr 2004; Uhr et al. 2008; Karlsson et al. 2011, 2013), no study to date has investigated the effect of chronic P‐gp inhibition on antidepressant distribution into the brain in wild‐type animals, to our knowledge. This is a key consideration, as currently available antidepressants are associated with a delayed response, typically requiring chronic treatment in order to achieve their therapeutic effect in patients (Krishnan and Nestler 2008; O'Leary et al. 2014). Moreover, certain xenobiotics are known to upregulate the expression and activity of P‐gp (Miller 2010). Indeed, the antidepressant venlafaxine, which is known to be a transported P‐gp substrate (O'Brien et al. 2012b), has been reported to increase P‐gp function in vitro and in vivo (Ehret et al. 2007; de Klerk et al. 2010). Thus, even though acute P‐gp inhibition can result in increased brain levels of certain antidepressants, this effect could be negated following chronic exposure due to a hypothesized counteractive upregulation of P‐gp activity.

The primary goal of the present study is to determine whether chronic treatment with the P‐gp inhibitor verapamil and the antidepressant escitalopram results in increased brain distribution of escitalopram. Recent studies, both in P‐gp knockout (acute and subchronic) (Karlsson et al. 2013) and wild‐type (acute only) (O'Brien et al. 2013b) rodents, have identified that escitalopram, a commonly prescribed selective serotonin reuptake inhibitor (SSRI) antidepressant, is a transported P‐gp substrate at the BBB. In addition, putative behavioral and molecular effects of chronic treatment with a P‐gp inhibitor and a P‐gp substrate antidepressant are investigated. In particular, the expression of several genes involved in the regulation of monoaminergic signaling, neurogenesis, responses to stress and gene transcription, and which have been reported to be sensitive to chronic antidepressant treatment, was assessed (Table 1).

**Table 1:** Target genes selected for mRNA expression analysis

Gene name	Protein product	Function	Studies implicating gene in antidepressant response
*Nr3c1*	Glucocorticoid receptor	Receptor for glucocorticoids, such as corticosterone in mice	Peiffer et al. (1991), Seckl and Fink (1992), Johansson et al. (1998), Bjartmar et al. (2000), Guidotti et al. (2013)
*Nr3c2*	Mineralocorticoid receptor	Cytosolic receptor for mineralocorticoids, such as aldosterone, as well as glucocorticoids	Brady et al. (1991), Seckl and Fink (1992), Johansson et al. (1998), Bjartmar et al. (2000)
*Fkbp5*	FK506‐binding protein	Immunophilin protein involved in immunoregulation and protein folding/trafficking	Guidotti et al. (2013)
*Egr1*	Early growth response protein 1 (aka Zif268 or NGFI‐A)	Transcription factor	Morinobu et al. (1997), Johansson et al. (1998), Bjartmar et al. (2000), Sillaber et al. (2008)
*Nr4a1*	Nerve growth factor IB	Transcription factor	Bjartmar et al. (2000)
*Slc6a4*	Serotonin transporter	Reuptake of 5‐HT from synaptic space	Lesch et al. (1993), Lopez et al. (1994), Benmansour et al. (1999)
*Tph2*	Tryptophan hydroxylase 2	Rate limiting enzyme in the synthesis of 5‐HT in CNS	Abumaria et al. (2007), Shishkina et al. (2007), Heydendael and Jacobson (2009)
*Htr1a*	5‐HT1A receptor	5‐HT autoreceptor involved in regulation of 5‐HT signaling	Burnet et al. (1994), Abumaria et al. (2007)
*Kcnk2*	Trek‐1	Potassium channel	Heurteaux et al. (2006)
*Bdnf*	Brain‐derived neurotrophic factor	Neurotrophin	Nibuya et al. (1995), Martinez‐Turrillas et al. (2005), Sillaber et al. (2008), Alboni et al. (2010)
*Creb*	cAMP response element‐binding protein	Transcription factor	Nibuya et al. (1996), Thome et al. (2000), Blom et al. (2002), Alboni et al. (2010)
*S100a10*	p11	Involved in regulation of 5‐HT signaling in brain	Svenningsson et al. (2006), Melas et al. (2012)

## Material and Methods

### Drugs and chemicals

Escitalopram oxalate was purchased from Discovery Fine Chemicals (Dorset, UK). Verapamil was obtained from Sigma‐Aldrich (Dublin, Ireland), as were all other chemicals, reagents and materials unless otherwise stated.

### Animals

Male C57BL/6JOlaHsd mice (6–8 weeks old at the beginning of the study), purchased from Harlan Laboratories, UK, were used in this study. All animals were group‐housed 4 animals per cage and maintained on a 12 h light/dark cycle (lights on at 08:00 h) with food and water ad libitum. Room temperature was controlled at 22 ± 1°C. All procedures were carried out in accordance with EU directive 2010/63/EU and approved by the Animal Experimentation & Ethics Committee of University College Cork.

### Experimental design

Verapamil (10 mg·kg^−1^ i.p.) or saline were administered at least 30 min before escitalopram (0.1 mg·kg^−1^ i.p.) or saline each morning for 22 days (*n* = 9–10 per group). Body weight was measured daily to monitor for potential adverse reactions to treatment. On the penultimate day of treatment (day 21), mice were subjected to the TST to assess the effect of chronic verapamil pretreatment on the antidepressant‐like activity of escitalopram. On the final day of treatment, mice were sacrificed 40 min after escitalopram and 100 min post‐verapamil administration. Timings were based on previous acute experiments (O'Brien et al. 2013b). Brain regions were immediately dissected out in ice‐cold PBS and trunk plasma was collected. All samples were snap frozen in isopentane on dry ice, and stored at −80°C until further processing. The dose of escitalopram was selected based on our previous study, where 0.1 mg·kg^−1^ of escitalopram was found to elicit a behavioral effect in the TST only when administered in conjunction with verapamil (O'Brien et al. 2013b). However, a lower dose of verapamil was used in the present study (10 mg·kg^−1^ rather than 20 mg·kg^−1^) due to concerns about potential systemic side effects that may have arisen with prolonged high dose verapamil treatment.

### Tail suspension test

The TST, one of the most widely used models for assessing antidepressant activity in rodents (Cryan et al. 2005), was carried out on the 21st day of treatment, as described previously (O'Brien et al. 2013b). This facilitated investigation of the impact of pretreatment with the P‐gp inhibitor verapamil on the antidepressant‐like activity of escitalopram after chronic administration of each drug. Briefly, on the day of the TST, the P‐gp inhibitor verapamil (10 mg·kg^−1^ i.p.) or saline was administered one hour before escitalopram (0.1 mg·kg^−1^ i.p.) or saline treatment. Thirty minutes after the second injection, mice were individually suspended by the tail from a horizontal bar using adhesive tape. Six‐minute test sessions were recorded by video camera and the amount of time spent immobile by each animal was subsequently scored by a trained observer blind to the treatment groups.

### Determination of escitalopram and verapamil in brain and plasma samples

Escitalopram and verapamil were extracted from cortical brain tissue and plasma using a liquid–liquid extraction technique described previously (Clarke et al. 2009; O'Brien et al. 2013b). Briefly, 48 *μ*L of plasma was spiked with 2 *μ*L of the internal standard, imipramine, to yield a final concentration of 1 *μ*g·mL^−1^ imipramine. To this imipramine‐spiked plasma, 1 mL of sodium hydroxide (2 mol/L) and 3 mL of water were added. Extraction was carried out in 7.5 mL of 1.5% isoamyl alcohol in *n*‐heptane by vortexing for 30 sec, followed by agitation on a mechanical shaker for 15 min and then centrifugation at 4500 g for 15 min at 20°C. The upper solvent layer was transferred to a tube containing 200 *μ*L of 25 mmol/L OPA, vortexed for 30 sec, then agitated on a mechanical shaker for 15 min followed by centrifugation at 5000 rpm for 15 min at room temperature. Twenty microlitres of the lower aqueous phase was injected onto a HPLC system for analysis of escitalopram and verapamil, using a previously described HPLC method (O'Brien et al. 2013b). Brain tissue samples were weighed prior to homogenization in 500 *μ*L of imipramine‐spiked (1 *μ*g·mL^−1^) homogenization buffer (i.e., HPLC mobile phase). Homogenized brain tissue was centrifuged at 35 280 g at 8°C for 15 min, and escitalopram and verapamil were extracted from 200 *μ*L of the supernatant as described above.

### P‐glycoprotein protein expression analysis

The effect of chronic verapamil and/or escitalopram treatment on the relative expression of P‐glycoprotein in hippocampal brain tissue was determined by Western blot (*n* = 7 per group), as described previously (O'Brien et al. 2013a), with some modifications. Protein was extracted from the hippocampus using a commercially available kit (*mir*Vana^™^ PARIS^™^ Kit; Applied Biosystems, Paisley, U.K.), as per the manufacturer's instructions. Protease and phosphatase inhibitor cocktails (Roche, Dublin, Ireland) were included in the tissue lysis buffer used in the extraction procedure. Sixteen micrograms of protein were loaded in a 4–20% gradient gel, according to the manufacturer's instructions (Express PAGE Gels; Genscript, Piscataway, New Jersey, US). After transfer onto a 0.2‐*μ*m nitrocellulose membrane and blocking with 5% skimmed milk and 0.1% Tween 20 in PBS, blots were probed overnight at 4°C with the C219 primary monoclonal P‐gp antibody (1:100 dilution in 2% skimmed milk) (Enzo Life Sciences (UK) Ltd, Exeter, UK). Reprobing was conducted for 1 h at room temperature using a goat anti‐mouse IgG‐HRP conjugate, diluted 1:2000 (Jackson Immunoresearch Europe Ltd, Suffolk, UK.). Images were obtained using a luminescent image analyzer (LAS‐3000; Fujifilm, Dublin, Ireland). For the detection of *β*‐actin, the membranes were incubated with Monoclonal Anti‐*β*‐Actin−Peroxidase antibody produced in mouse (1:15,000). Immunoblots were quantified using ImageJ software (http://imagej.nih.gov/ij/, National Institute of Health, Maryland, USA).

### Gene expression analysis

The hippocampal expression of twelve genes, which have been shown to be responsive to chronic antidepressant treatment or implicated in the antidepressant response (Table 1), was analyzed using real‐time quantitative polymerase chain reaction (qPCR). To our knowledge, p11, brain‐derived neurotrophic factor (BDNF) and cAMP‐responsive element‐binding protein (CREB) are the only gene targets which have been shown to be altered by chronic escitalopram treatment in the (rat) brain (Jacobsen and Mork 2004; Alboni et al. 2010; Melas et al. 2012). Total RNA was extracted from hippocampal tissue using a commercially available kit (*mir*Vana^™^ PARIS^™^ Kit, Applied Biosystems), as per the manufacturer's instructions. RNA purity and quantity was measured by spectrophotometric analysis (NanoDrop^®^ ND‐1000 Spectrophotometer, NanoDrop Technologies, Inc., Wilmington, Delaware, USA). mRNA was reverse transcribed from 1 *μ*g total RNA using a G‐storm thermocycler (G‐storm, Surrey, UK). PCR primers and probes were purchased from Integrated DNA Technologies (Leuven, Belgium). Assays were designed for murine genes, as detailed in Table 1. Analysis of gene expression was performed in triplicate on 384‐well plates using the LightCycler 480 System (Roche), and the expression of each gene was normalized to that of *β*‐actin. The 2^−ΔΔCT^ method was used to calculate relative changes in gene expression determined from qPCR experiments (Livak and Schmittgen 2001). Gene expression values are expressed relative to the control group.

### Data analysis and statistical procedures

Statistical analysis of data was carried out using standard commercial software (SPSS Statistics, version 20.0.0; SPSS, Inc., Chicago, IL). The differences in brain and plasma levels of escitalopram between the two escitalopram‐treated groups were analyzed using an unpaired Student's *t*‐test. Similarly, verapamil concentrations in brain tissue and plasma were compared by unpaired Student's *t*‐test. Differences in gene expression between the groups were analyzed by two‐way analysis of variance (ANOVA), with verapamil and escitalopram as factors, and the LSD post hoc was used to elucidate statistically significant differences between treatment groups. Differences in changes in body weight over time were analyzed by two‐way repeated measures ANOVA, with escitalopram and verapamil as factors. The criterion for statistical significance was *P *<* *0.05.

## Results

### Concomitant chronic treatment with the P‐gp inhibitor verapamil and escitalopram increased the brain levels of escitalopram, without affecting plasma levels

**Figure 1:**
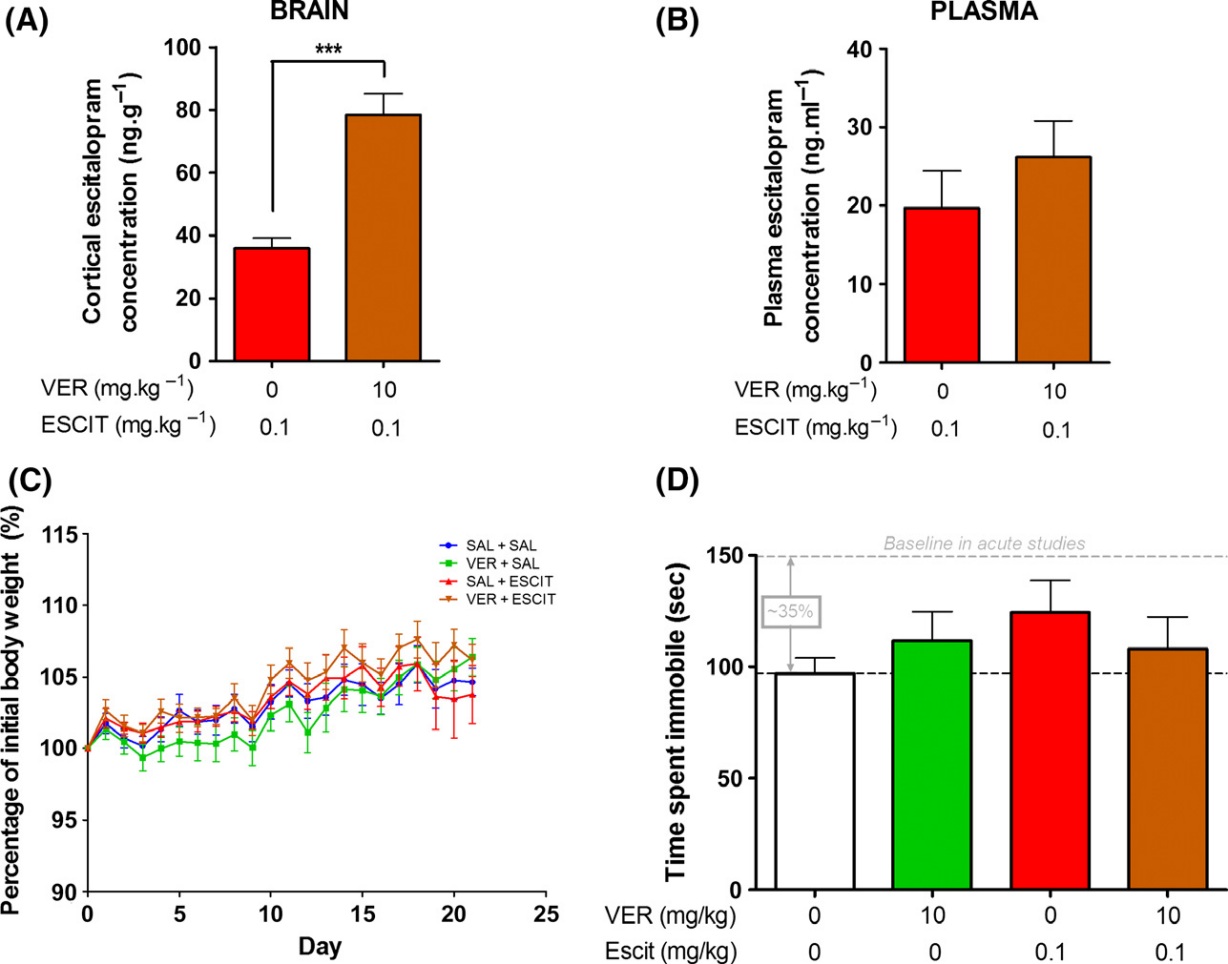
Effect of chronic administration of verapamil and escitalopram on brain concentrations of escitalopram, body weight and antidepressant‐like behavioral effects in the tail suspension test (TST). (A) Pretreatment with verapamil resulted in a 110% increase in concentrations of escitalopram in cortical brain tissue. (B) Pre‐treatment with verapamil did not significantly alter plasma levels of escitalopram. (C) Treatment with verapamil and/or escitalopram had no effect on body weight compared to the saline‐treated control group. (D) There were no statistically significant differences between the treatment groups in terms of immobility in the TST. However, it it worth noting that the baseline immobility of the saline‐treated control group was reduced by ~35% compared to our previous acute work (O'Brien et al. 2013b). (*n* = 9–10 per group). ***P < 0.001 between groups.

The concentrations of escitalopram in cortical brain tissue were significantly greater in mice pretreated with the P‐gp inhibitor verapamil compared to saline‐pretreated mice, whereas there was no significant difference in plasma escitalopram levels (Fig. 1A and B). Pretreatment with verapamil resulted in a 110% increase in cortical escitalopram levels (*t*(17) = −5.903, *P *<* *0.001; Fig. 1A), but had no effect on plasma levels of escitalopram (*t*(17) = −0.98, *P *=* *0.341); Fig. 1B). Thus, the increase in brain concentrations of escitalopram can be attributed to enhanced BBB transport due to P‐gp inhibition, rather than a reflection of elevated plasma concentrations.

Plasma (*t*(17) = 0.274, *P *=* *0.787) and brain (*t*(14) = −0.810, *P *=* *0.937) levels of verapamil were equivalent in both verapamil‐treated groups, with mean (±SEM) concentrations of 390 (±37) ng·mL^−1^ in plasma and 202 (±15) ng·g^−1^ in cortical tissue.

### Body weight

Neither drug treatment, nor a combination of both drugs, had an effect on body weight during the course of the 22 day of drug administration (Fig. 1C): escitalopram (*F*(1,35) = 0.376, *P *=* *0.544); verapamil (*F*(1, 35) = 0.096, *P *=* *0.759); and escitalopram × verapamil (*F*(1, 35) = 0.195, *P *=* *0.661).

### Tail suspension test

Neither escitalopram (*F*(1, 35) = 0.885, *P *=* *0.353) nor verapamil (*F*(1, 35) = 0.004, *P *=* *0.950) had a significant impact on the duration of immobility in the TST, nor was there an interaction between the two factors (*F*(1, 35) = 1.505, *P *=* *0.228) (Fig. 1D). In comparison to previously reported acute experiments performed in our laboratory (O'Brien et al. 2013b), chronic administration reduced the time spent immobile in saline‐treated mice by 35% (97s vs. 150s).

### P‐glycoprotein expression analysis

**Figure 2:**
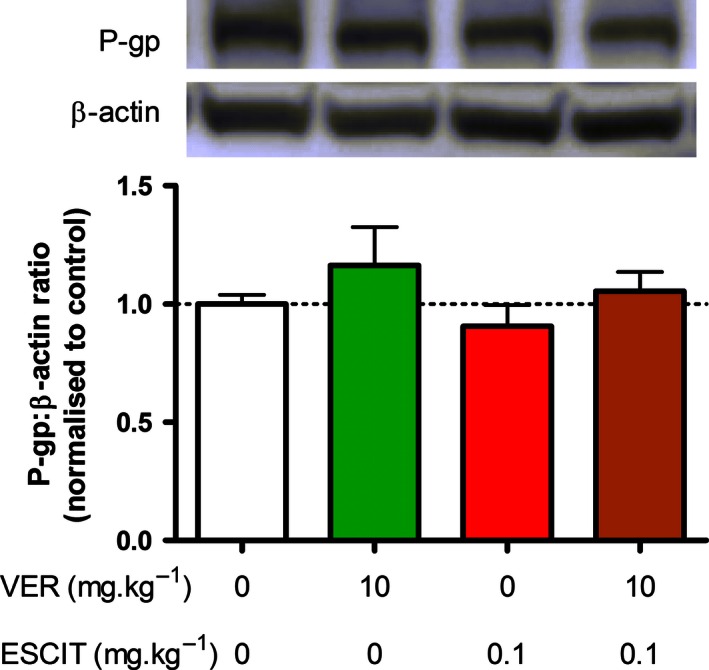
Western blot analysis of P‐gp protein expression in the hippocampus. Chronic administration of verapamil and/or escitalopram elicited no effect on the expression of P‐gp protein in hippocampal tissue (*n* = 7 per group).

Hippocampal P‐gp protein levels were unaffected by chronic treatment with either verapamil, escitalopram, or a combination of both drugs (Fig. 2). This indicates that P‐gp expression was neither upregulated nor downregulated in response to repeated long‐term administration of this P‐gp inhibitor or P‐gp substrate antidepressant.

### Gene expression analysis

A subset of animals was used for gene expression analysis. The total numbers of samples included for each gene are listed in Table 2. Two‐way ANOVA analysis of PCR results revealed a verapamil, escitalopram or verapamil × escitalopram effect on the expression of four of the twelve genes analyzed (Table 2).

**Table 2:** Two‐way ANOVA of the effects of verapamil, escitalopram and verapamil × escitalopram interaction on mRNA expression in the hippocampus (*n* = 7–10 per group)

Target	Total n	Verapamil effect	Escitalopram effect	Verapamil × escitalopram interaction
GR	32	*F*(1, 28) = 0.15, *P *=* *0.904	*F*(1, 28) = 4.132, *P *=* *0.052	*F*(1, 28) = 2.337, *P *=* *0.138
MR	31	*F*(1, 27) = 1.530, *P *=* *0.227	*F*(1, 27) = 0.004, *P *=* *0.952	*F*(1, 27) = 0.059, *P *=* *0.810
FKBP5	34	*F*(1, 30) = 0.810, *P *=* *0.375	*F*(1, 30) = 0.669, *P *=* *0.420	*F*(1, 30) = 1.385, *P *=* *0.249
EGR1	34	*F*(1, 30) = 3.795, *P *=* *0.061	***F*** **(1, 30) = 5.854, ** ***P *** **=** *** *** **0.022**	*F*(1, 30) = 0.011, *P *=* *0.917
NGFI‐B	31	***F*** **(1, 27) = 5.336, ** ***P *** **=** *** *** **0.029**	*F*(1, 27) = 1.838, *P *=* *0.186	*F*(1, 27) = 0.389, *P *=* *0.538
SERT	33	*F*(1, 29) = 0.408, *P *=* *0.528	*F*(1, 29) = 0.009, *P *=* *0.925	***F*** **(1, 29) = 5.385, ** ***P *** **=** *** *** **0.028**
TPH2	33	*F*(1, 29) = 0.284, *P *=* *0.598	*F*(1, 29) = 1.258, *P *=* *0.271	*F*(1, 29) = 3.342, *P *=* *0.078
5‐HT1A	33	*F*(1, 29) = 0.008, *P *=* *0.930	*F*(1, 29) = 1.091, *P *=* *0.305	*F*(1, 29) = 0.414, *P *=* *0.525
TREK‐1	33	*F*(1, 29) = 1.568, *P *=* *0.221	*F*(1, 29) = 0.091, *P *=* *0.765	*F*(1, 29) = 2.216, *P *=* *0.147
BDNF	34	*F*(1, 30) = 0.000, *P *=* *0.990	*F*(1, 30) = 0.918, *P *=* *0.346	*F*(1, 30) = 0.062, *P *=* *0.805
CREB	32	*F*(1, 28) = 2.163, *P *=* *0.153	*F*(1, 28) = 0.008, *P *=* *0.930	*F*(1, 28) = 0.477, *P *=* *0.496
p11	34	***F*** **(1, 30) = 10.182, ** ***P *** **=** *** *** **0.003**	*F*(1, 30) = 0.076, *P *=* *0.785	*F*(1, 30) = 0.127, *P *=* *0.724

Bold font denotes statistically significant effect.

GR, glucocorticoid receptor; MR, mineralocorticoid receptor; FKBP5, FK506‐binding protein; EGR1, early growth response protein 1; NGFI‐B, nerve growth factor IB; SERT, serotonin transporter; TPH2, tryptophan hydroxylase 2; BDNF, brain‐derived neurotrophic factor; CREB, cAMP response element‐binding protein.

**Figure 3:**
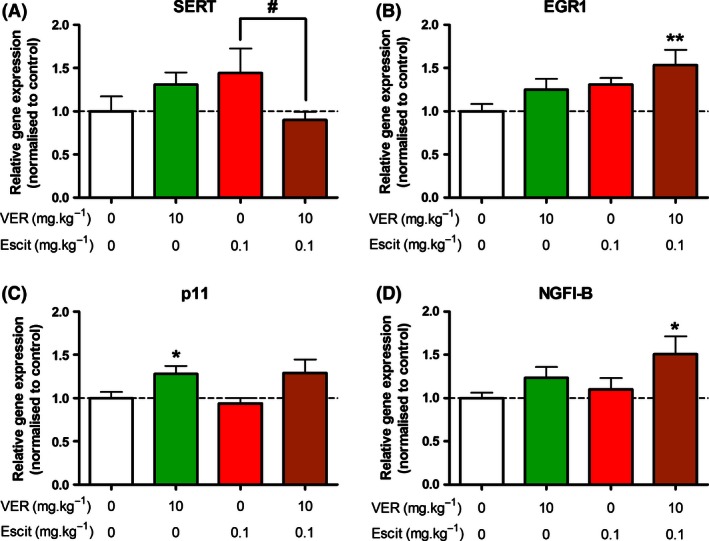
Relative hippocampal expression of genes for which significant effects were observed. (A) A significant interaction between verapamil and escitalopram was observed for SERT expression. SERT mRNA levels were significantly lower in mice treated with both verapamil and escitalopram than those treated with escitalopram only. (B) Escitaloram treatment was found to exert a significant effect on EGR1 mRNA expression. The increase in EGR1 mRNA levels, compared to the control group, was only statistically significant in mice treated with both verapamil and escitalopram. However, there was no significant interaction between the two treatments. (C) Verapamil exerted a significant effect on p11 expression, resulting in a significant increase in mice treated with verapamil only compared to control mice, and a trend toward increased expression in mice treated with both verapamil and escitalopram (*P *=* *0.054). (D) Verapamil also exerted a significant effect on NGFI‐B expression. A statistically significant elevation in NGFI‐B mRNA was only observed in mice treated with both verapamil and escitalopram, but there was no interaction between the two treatments (*n* = 7–10 per group). *P < 0.05; **P < 0.01 compared to control group. ^#^ *P* < 0.05 between the indicated groups.

The only gene expression target for which a significant escitalopram × verapamil interaction was detected was the serotonin transporter (SERT). Chronic treatment with verapamil or escitalopram individually resulted in a trend toward increased SERT mRNA expression, but this trend was reversed when both verapamil and escitalopram were administered together, resulting in a significant reduction in SERT mRNA levels compared to mice treated with escitalopram only (Fig. 3A).

Escitalopram treatment had an effect on the expression of early growth response protein 1 (EGR1) mRNA, resulting in increased expression of this gene. Post hoc analysis revealed that this increase was only statistically significant in mice treated with both verapamil and escitalopram. However, there was no significant interaction between the two treatments (Table 2; Fig. 3B).

Verapamil treatment affected the expression of mRNA for p11 and nerve growth factor IB (NGFI‐B), increasing p11 expression to a similar extent with or without escitalopram treatment (Fig. 3C). NGFI‐B mRNA expression was also increased by verapamil treatment. Similar to EGR1, post hoc analysis revealed that this increase was only statistically significant in mice treated with both verapamil and escitalopram, but there was no significant interaction between the two treatments (Table 2; Fig. 3D).

## Discussion and Conclusions

Recent evidence from preclinical studies has highlighted an important role for the efflux transporter P‐gp at the BBB in limiting the brain distribution of several antidepressants (O'Brien et al. 2012b). Here, it is shown for the first time, to our knowledge, that chronic treatment with a P‐gp inhibitor and a P‐gp substrate antidepressant results in increased brain levels of the antidepressant drug. We recently demonstrated that acute pre‐treatment with the P‐gp inhibitor verapamil augmented the brain concentrations and functional activity of the SSRI escitalopram in mice (O'Brien et al. 2013b). In addition, in vitro bidirectional transport studies demonstrated that escitalopram is a transported substrate of human P‐gp (O'Brien et al. 2013b), indicating that these pharmacokinetic and pharmacodynamic observations in mice may translate to man. However, prior to the present study, it remained unclear if acute findings would apply to chronic treatment paradigms. Considering that antidepressant drugs are administered chronically in clinical practice, this is an important point, especially given that another P‐gp substrate antidepressant (venlafaxine) has been reported to induce P‐gp in vitro and in vivo (Ehret et al. 2007; de Klerk et al. 2010). Thus, the present results indicate that P‐gp inhibition may represent a promising strategy to augment the brain delivery of certain antidepressants during chronic treatment.

The greater than 2‐fold increase in brain concentrations of escitalopram in mice treated with the P‐gp inhibitor verapamil in the present chronic study is consistent with previously observed effects in an acute setting (O'Brien et al. 2013b), despite using a lower dose of verapamil in the present work (10 mg/kg daily vs. 20 mg/kg previously). However, in contrast with the acute study, no enhancement of behavioral or gene expression responses to chronic escitalopram treatment was detected here. Indeed, chronic treatment with escitalopram elicited no robust effect on the behavioral or molecular parameters investigated in the present study. This is perhaps unsurprising, given that a relatively low dose (0.1 mg·kg^−1^) of escitalopram was chosen so as to be able to unmask any augmentation effects of verapamil.

In previous acute studies, we had observed a behavioral effect in the TST at this low dose of escitalopram only when mice were pretreated with verapamil (O'Brien et al. 2013b). No such effect was evident here after chronic administration of both drugs. However, when compared to our previous acute experiments (cf. O'Brien et al. 2013b), the baseline immobility in the control group in this chronic study was reduced by 35% (Fig. 1D), indicating that repeated daily handling and injections impacted on the behavioral readout of the TST. This may have resulted in a floor effect, thereby potentially obscuring any putative behavioral impact of the increased brain concentrations of escitalopram due to P‐gp inhibition and rendering the results from the TST inconclusive. Although there have been a limited number of reports of the TST being responsive to chronic antidepressant administration, it is predominantly used to assess behavioral responses to acute antidepressant treatment (Cryan et al. 2005). Interestingly, studies which have yielded positive results in the TST following chronic antidepressant administration have generally involved quite high doses of the antidepressant, for example 20 mg·kg^−1^ of fluoxetine (Ukai et al. 1998). Hence, increasing the dose of escitalopram may represent a viable strategy to overcome the floor effect seen in the TST here in future. In addition, it will be important to validate the antidepressant potential of esciatlopram in combination with verapamil in alternative antidepressant testing paradigms, such as the social defeat stress or learned helplessness models (O'Leary and Cryan 2013), as these tests are responsive to chronic antidepressant treatment and have the added advantage of being mouse models of depression, as opposed to the present study which involved ‘normal’ mice.

Similar to our behavioral studies, gene expression analysis did not reveal an augmentation of molecular responses to escitalopram administration in verapamil pretreated mice. However, reports of alterations in mRNA expression in response to antidepressant treatment are often inconsistent, and may not be universal among different antidepressants, as evidenced by the variable findings in relation to BDNF expression (Tardito et al. 2006; Groves 2007). Thus, genes which have been reported to be altered by treatment with other antidepressants may not generalize to escitalopram administration. To our knowledge, p11, BDNF and CREB are the only gene targets which have been shown to be affected by chronic escitalopram treatment (Jacobsen and Mork 2004; Alboni et al. 2010; Melas et al. 2012). However, the expression of these genes was not altered by escitalopram treatment in the present study, which may have been due to the 100‐fold lower dose of escitalopram used here than in previous work (0.1 mg/kg per day compared to 10 mg/kg per day). Moreover, our finding that chronic escitalopram treatment exerted a significant effect on mRNA levels of the transcription factor EGR1 has not previously been reported, to our knowledge. Interestingly, the combination of verapamil and escitalopram treatment, but neither in isolation, resulted in a statistically significant increase in EGR1 mRNA expression relative to control mice. Similar findings were observed in relation to another transcription factor, NGFI‐B. While there was not a significant interaction between escitalopram and verapamil treatment for either of these genes, indicating that this could merely reflect an additive effect of the two treatments, these targets warrant further investigation in future studies.

It is also worth noting that antidepressant‐induced changes in gene expression can be dependent on the brain region analyzed and the antidepressant dose used (Tardito et al. 2006). In relation to the dose, the drug administration regimen used in the present study yielded plasma concentrations of escitalopram (23 ng·mL^−1^) within the therapeutic range (15–80 ng·mL^−1^) (Baumann et al. 2004), albeit at the lower end of that range. However, most studies which have reported significant alterations in mRNA expression following antidepressant treatment have involved the administration of much higher doses of the antidepressant (10 mg·kg^−1^ escitalopram daily, for example (Jacobsen and Mork 2004; Alboni et al. 2010)). Thus, the doses of escitalopram used in the present study may have been insufficient to reproduce previously reported changes in mRNA expression. Furthermore, it is possible that there may have been functionally relevant alterations in protein expression, activation or cellular localization in response to antidepressant treatment, without changes in mRNA levels. Future studies should investigate these possibilities.

Even though our analysis did not reveal any augmentation of escitalopram's effects on mRNA expression by verapamil administration, a statistically significant interaction between the two treatments was observed in terms of serotonin transporter (SERT) mRNA levels. Interestingly, this effect constituted an attenuation of a trend toward increased SERT mRNA levels following verapamil or escitalopram on their own. It is important to note that, while studies have shown that SERT mRNA expression can be altered in response to antidepressant treatment, these findings have been somewhat inconsistent. SERT mRNA levels have been variously reported to be increased, decreased or not affected by chronic antidepressant treatment (Lesch et al. 1993; Lopez et al. 1994; Benmansour et al. 1999; Abumaria et al. 2007), indicating that the effect of antidepressant drugs on SERT expression is complex and variable.

Due to the inconclusive nature of our findings in relation to the effect of chronic P‐gp inhibition on behavioral and molecular responses to antidepressant treatment, further studies are required to investigate this question. Such future studies should ideally utilize more specific inhibitors of P‐gp, thereby limiting the contribution of P‐gp independent factors to alterations in gene expression, and helping to specifically elucidate antidepressant‐related changes. For example, two‐way ANOVA analysis revealed that verapamil itself exerted a significant effect on the expression of mRNA for NGFI‐B and p11, thus making it difficult to identify any potential influence of P‐gp inhibition on putative escitalopram effects in relation to these targets. In addition, future studies should use different dosing regimens of escitalopram and investigate other parameters, such as protein expression, rather than focusing on mRNA expression exclusively.

Verapamil, the P‐gp inhibitor used in the present study, is a clinically used calcium channel blocker, indicated for the treatment of several cardiovascular conditions. At higher doses, which it is thought may be required to inhibit P‐gp due to its lack of specificity and relatively low potency, verapamil can elicit toxicity (DeWitt and Waksman 2004). In the present study, in which verapamil was found to inhibit P‐gp, no overt signs of toxicity were detected during daily observations or in terms of alterations in body weight. Furthermore, the plasma concentrations of verapamil measured in the present study (390 ng·mL^−1^) are within the clinical therapeutic range (50–500 ng·mL^−1^) (Regenthal et al. 1999). However, it is likely that its other pharmacological actions, including cardiovascular effects and inhibition of cytochrome P450 enzymes, would preclude verapamil's widespread use as a P‐gp inhibitor in clinical practice. Several more specific second and third generation P‐gp inhibitors have been developed which may be more appropriate for this purpose (Colabufo et al. 2010). Nonetheless, verapamil represents a valuable pharmacological tool to investigate the effects of P‐gp inhibition on drug distribution in preclinical studies. Another important consideration in this regard is the possibility that certain antidepressants, such as clomipramine, have been reported to influence P‐gp function in vitro (Pariante et al. 2003; Weiss et al. 2003), even if the in vivo relevance of these findings remains unclear (Mason et al. 2011). Further studies are required to add clarity to this issue.

In conclusion, the present study demonstrates that inhibition of P‐gp results in elevated brain levels of an antidepressant drug in a chronic drug administration paradigm. This finding indicates that P‐gp inhibition may represent a viable strategy to enhance the brain distribution of P‐gp substrate antidepressants after repeated dosing. In addition, these data reveal that chronic escitalopram administration can influence mRNA expression of the transcription factor EGR1. Further studies are required to fully elucidate the functional consequences of increasing the brain levels of an antidepressant drug by P‐gp inhibition on a prolonged basis.

## Disclosures

The authors declare no conflict of interest.
